# Changes in the expression of cardiac genes responsive to thyroid hormones in the chickens with cold-induced pulmonary hypertension

**DOI:** 10.1016/j.psj.2021.101263

**Published:** 2021-05-25

**Authors:** S. Bahadoran, H. Hassanpour, S. Arab, S. Abbasnia, A. Kiani

**Affiliations:** ⁎ Department of Clinical Sciences, Faculty of Veterinary Medicine, Shahrekord University, Shahrekord, 34141-88186 Iran; † Department of Basic Sciences, Faculty of Veterinary Medicine, Shahrekord University, Shahrekord, 34141-88186 Iran

**Keywords:** thyroid hormones, ascites, congestive heart failure, contractile elements

## Abstract

Cold stress is an environmental cause of pulmonary hypertension syndrome (**PHS**) in broiler chickens. This factor could increase the rate of metabolic activity via thyroid hormones (T3 and T4). To evaluate the effect of these hormones on the heart, the plasma concentration of T3, T4, and the gene expression of their receptors (THRα and THRβ) and many contractile proteins (ACTC1, MHCα, MHCβ, RYR2, SERCA2, THRα, THRβ, and troponin I) were measured in the right ventricle in 2 periods of age (21 and 35 d). Plasma T3 concentration was significantly higher in the PHS group of chickens than in the control one at 21 and 35 d while plasma T4 did not change. The relative expression of MHCα, RYR2, SERCA2, and THRα genes in the right ventricle tissues was only higher in PHS group of broilers than control group at 21 d (*P* < 0.05) whereas the expression of ACTC1, MHCβ, and troponin I did not differ at 2 periods of age. The positive correlations between MHCα, RYR2, SERCA2, and T3, THRα were confirmed. The expression of THRβ gene was only higher in PHS group of broilers than control at 35 d (*P* < 0.05). The data determined that cold stress could increase thyroid hormones and the gene expression of their receptor (THRα) in the pick of chicken growth (21 d) that they themselves elevates the expression of many genes related to contractile elements (MHCα, RYR2, and SERCA2), leading to adaptive right ventricle hypertrophy.

## INTRODUCTION

Low ambient temperature is one of the critical stressors for fast-growing broiler chickens. Prolong cold stress could stimulate the hypothalamic – pituitary – adrenal/thyroid axis and increase stress responses by hormones such as adrenaline, cortisone and thyroxine (**T4**) and triiodothyronine (**T3**) (Hangalapura et al., 2004). Thyroid hormones (T3 and T4) enhance cellular metabolism that in itself elevate cellular consumption of oxygen and production of carbonic dioxide (Kahaly and Dillmann, 2005). To compensate, the pulmonary circulation and right ventricle of heart are overloaded to exchange more gas in the lung. The rigid lung and insufficient development of its vasculature limits the gas exchange in the chickens leading to severe hypoxia that it increases pulmonary blood flow/arterial pressure and right ventricular afterload in a positive feedback. This cascade process has been known as pulmonary hypertension syndrome (**PHS**) with clinical lesions of pericarditis, right ventricular hypertrophy/dilation, liver congestion, and ascites (Scicchitano et al., 2016; Biswas, 2019).

In addition to basal metabolism, thyroid hormones affect the cardiomycytes to initially increase myosin^٬^s power stroke of contraction, cardiac output and then to rise blood supply in the tissues with high metabolic rate (Vale et al., 2019). Thyroid hormones have 2 extranuclear and nuclear effects in the cardiomycytes. Extranuclear effect is associated with the protein synthesis and upregulation of the membrane transporters and channels, while nuclear effect of thyroid hormones that is mediated by thyroid receptors (THRα and THRβ), regulates transcription of cardiac genes responsive to thyroid hormones (Kahaly and Dillmann, 2005; Grais and Sowers, 2014). The mRNA of these cardiac genes codes several proteins that directly or indirectly contribute in the myocardial contractility. In mammals, it has been determined that thyroid hormones may upregulate genes such as sarcoplasmic reticulum calcium adenosine triphosphatase (**SERCA2**, transporter of calcium from the cytosol into the sarcoplasmic reticulum), myosin heavy chain α (**MHCα**, major protein of the cardiac muscle thick filament), Alpha cardiac actin (**ACTC1**, major protein of the cardiac muscle thin filament), troponin I (a protein that prevents myosin from binding to actin in relaxed muscle), ryanodine receptor 2 (**RYR2**, major mediator for sarcoplasmic release of stored calcium ions) and downregulate the mRNA of genes such as myosin heavy chain β (**MHCβ**, a protein with the enzymatic activity of the ATPase in the myosin head), Phospholamban (regulator of the calcium pump), sodium/calcium exchanger, and Adenylyl cyclase genes. All these alterations are in direction to potentiate the cardiac contraction in the early hyperthyroidism, while in the progressive hyperthyroidism that cellular catabolism occurs, the gene regulation of thyroid hormones inversely changes, leading to cardiac contractile dysfunction.

In this study, our aim is to investigate the alteration of cardiac gene expression (ACTC1, MHCα, MHCβ, RYR2, SERCA2, THRα, THRβ, and troponin I) responsive to thyroid hormones in the broiler chickens exposed to low temperature that progressively indicated the signs of PHS and ascites (Davis and Davis, 2002; Dillmann, 2002; Kahaly and Dillmann, 2005).

## MATERIALS AND METHODS

### Bird Management and Induction of Pulmonary Hypertension by Cold Stress

All the procedures employed in this experiment were approved by the Institutional Animal Care and Use Committee of Shahrekord University which was in accordance with the standard of 1964 Declaration of Helsinki. A total of 54 one-day-old male broiler chicks (Ross 308) were selected to have narrow weight range and randomly divided to 2 groups (control and treatment) to have 27 birds per group. Each group was housed in a separate room, and assigned into 3 equal replicates of 9 chickens per pen (3 pen/room with size of 1.5 × 1.5 m). Chickens were raised on wood shavings under standard conditions for 35 d outlined by the manufacturer (Ross 308 guideline), and provided free access to water and a standard ration (according to Ross 308 recommendations) by water and feed dispensers. In the room of treatment group, gradual low temperature (as cold stress) was applied during rearing period according to Teshfam et al. (2006) and Hassanpour et al. (2016b) to induce pulmonary hypertension (Table 1). Throughout the rearing period, mortality was recorded daily, and dead broilers were examined for lesions of ascites, especially heart failure.

**Table 1:** Room temperatures (°C) during the experimental period.

	Age (d) of chickens											
Experimental groups	1	3	5	7	9	11	13	15	17	19	21	23–35
Control	32	31	30	29	28	27	26	25	24	23	22	21–22
Cold-induced PHS	32	31	30	29	25	22	19	18	17	16	16	14–15

### Blood and Heart Tissue Sampling and Estimation of Pulmonary Hypertensive Index

At 21 and 35 d, 12 chickens from each experimental group (5 birds/pen) were randomly selected for blood collection and processing. Blood samples (~ 3 mL) were collected from brachial vein and centrifuged at 2,500 × *g* for 10 min to separate sera. Those birds were then decapitated, and their hearts were harvested. According to Hassanpour et al. (2009), heart ventricles were dissected from vessels/atria and weighed. Then, the right ventricular‐to‐total ventricular weight ratio (**RV:TV** ratio) was calculated. As described by Wideman (2001), Whenever RV:TV exceeded 0.29, it was considered as an indication of PHS in the broiler chickens. Morbidity of PHS is determined after killing of all chickens according to this ratio. The tissues were immediately frozen in liquid nitrogen, and stored at −70°C until RNA extraction

### The Measurement of Thyroid Hormones

Total T3 and T4 were determined on plasma samples by performing radioimmunoassay, using commercial kits (Institute of Isotopes Ltd., Budapest, Hungary) and a gamma counter (Genesys Gamma; Laboratory Technologies Inc., Elburn, IL). The T3 assay was characterized by intraassay and interassay coefficient of variations (**CV**) of 0.3 nm/l and 4.7%, respectively. The T4 assay was characterized by intraassay and interassay CV of 7.0 nm/l and 6.3%, respectively.

### Total RNA Isolation, DNAase Treatment, and cDNA Synthesis

To extract total RNA, the right ventricle tissue (100 mg) of 12 birds per treatment were harvested, homogenized in liquid nitrogen under aseptic conditions and treated by a digestion buffer containing guanidine and phenol (RNX-PLUS solution, Sinaclon Bioscience, Karaj, Iran). After adding chloroform to the digested samples and centrifuged at 6,000 *g* and 4°C for 15 min, the upper aqueous phase of supernatant was separated. The supernatant was taken to precipitate total RNA using the equal volume of isopropanol (100%) and centrifuged (6,000 *g*, 4°C, 10 min). The visible pellet were then rinsed with 75% ethanol, centrifuged, and the resulting pellet dissolved in appropriate amount of diethyl dicarbonate (**DEPC**)‐treated water (Ahmadipour et al., 2015; Pirany et al., 2020).

The extracted RNA was treated with DNAase kit (Sinaclon Bioscience, Karaj, Iran) to remove genomic DNA contamination. The early qualification test for the extracted RNA was to observe discrete 18S and 28S rRNA bands onto agarose gel (1.5%) of electrophoresis. The quantity of RNA was checked by O.D. (A260/280) using a biophotometer (Eppendorf, Hamburg, Germany). RNA with the absorbance ratio between 1.8 and 2.2 was used for the synthesis of cDNA (Hassanpour et al., 2015a).

Total RNA (1 *μ*g) was converted to cDNA by means of the PrimeScript RT Reagent Kit (Takara Bio Inc., Japan). The synthesized cDNA was then stored at *−*20°C (Ahmadipour, et al., 2015).

The levels of ACTC1, MHCα, MHCβ, RYR2, SERCA2, THRα, THRβ, and troponin I and tyrosine 3‐monooxygenase/tryptophan 5‐monooxygenase activation protein zeta (**YWHAZ**) transcripts were determined by real‐time RT‐PCR using SYBR® Premix Ex Taq II kit (TliRnase H Plus; Takara Bio Inc., Otsu, Japan). YWHAZ was used as an endogenous reference gene to normalize the input load of cDNA across samples (Hassanpour et al., 2018). Table 2 represents the specific primers of PCR, designed with the online software of Primer‐Blast (www.ncbi.nlm.nih. gov/tools/primer-blast/index.cgi?LINK_LOC=BlastHome).

**Table 2:** Primers used for quantitative real time PCR analysis of chicken mRNAs.

Gene	Primer sequence (5′-3′)	GC (%)	Tm (°C)	PCR Product (bp)	Accession No.
ACTC1	TGTGACGACGAGGAGACCAC	60	61.51	110	NM_001079481.1
	CCGACGATGGATGGGAACAC	60	60.81		
MHCα	CAGGTGCAATCCCAGAGGAC	60	60.39	246	NM_204766.2
	CCCTGCGCTCCACTATCTTC	60	60.25		
MHCβ	GAGGAGGAGTGCATGTTCCC	60	60.11	148	NM_001001302.1
	CCGCGTAGTGGATGAGTGAG	60	60.25		
RYR2	CCCTGAAGCTAGGGATTGCC	60	60.18	199	XM_025149046.1
	CCCTCTTCAGTCACCATGCC	60	60.39		
SERCA2	GTCTTCCCTCCGTGGAAACC	60	60.32	170	M66385.1
	GGGAGCGTATGTCGAACCAG	60	60.53		
THRα	AGACGTACCTGCTGGCGTTC	60	62.49	150	NM_205313.2
	GGCACTCCACCTTCATGTGC	60	61.59		
THRβ	GGCCTTGTTTGCGTCGAGAG	60	61.90	187	X17504.1
	GGCACTCCACCTTCATGTGC	60	61.59		
Troponin	TGGATGAGGAGCGCTACGAC	60	61.73	261	NM_213570.1
	CCACGTTCTTCCGCCAATCG	60	61.97		
YWHAZ	AGGAGCCGAGCTGTCCAATG	60	62.25	83	NM-001031343.1
	TCCAAGATGACCTACGGGCTC	57	61.30		

**Figure 1:**
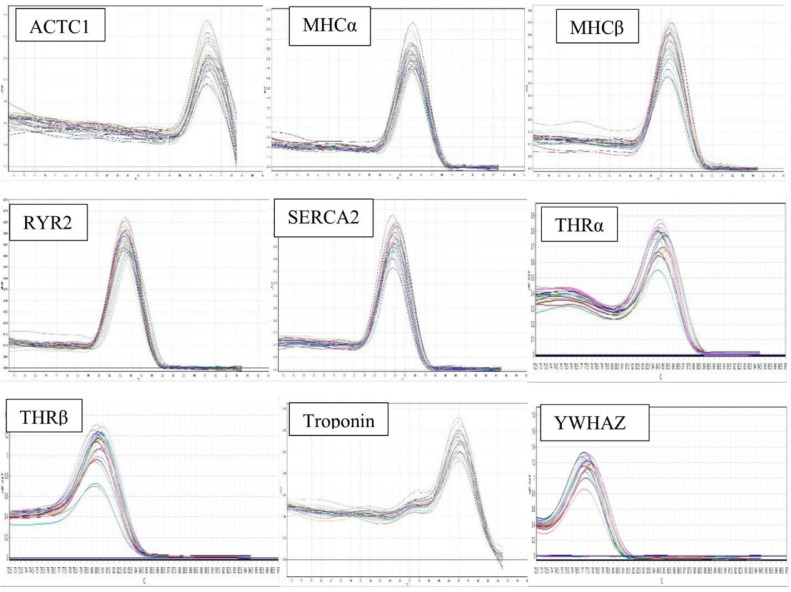
Specificity of real-time PCR amplification. Melting curves (dissociation curves) of the 9 reference gene amplicons after the real-time PCR reactions, all showing 1 peak. X-axis (horizontal): temperature (°C); Y-axis (vertical): negative derivative of fluorescence over temperature (–dF/dT).

The amplifications were run by a real-time thermocycler (Rotor Gene Q 6000, Qiagen, Valencia, CA) for each sample in 3 replicates. The PCR program was initial 95°C for 2 min followed with 35 to 40 cycles of 95°C for 15 s, and 60 to 62°C for 30 s. The melt curve of each applicant was assessed for the specificity of primers/amplicons and the absence of primer dimer formation (Figure 1). LinRegPCR software version 2012.0 (Amsterdam, Netherlands) was applied to analyze threshold cycle number values and calculate mean efficiency value for each gene (Ruijter et al., 2009). Relative transcript levels (gene/YWHAZ) were calculated using the efficiency‐adjusted Paffl methodology (Hassanpour et al., 2015b).

### Statistical Analysis

Differences between 2 experimental group means were analyzed through independent Student's *t* test with SPSS 26.0 (SPSS, Chicago, IL). All results are shown as mean ±SE and differences between two control and PHS groups of broilers were considered significant at *P <* 0.05. The correlation between MHCα, RYR2, SERCA2, and T3, THRα was evaluated using nonlinear regression.

## RESULTS

### Pulmonary Hypertensive Index

As an index of cold-induced pulmonary hypertension at 21 and 35 d of age, the RV:TV ratio was higher in the heart samples of chickens exposed to low temperature compared to healthy chickens (*P <* 0.05; Table 3). The clinical lesions of PHS were mostly hydropericarditis, enlargement of heart and liver in the cold-treated chickens (as PHS group) at 21 d while the accumulation of fluid in the abdominal cavity was also predominance at 35 d. The overall morbidity and mortality rates were 74 and 14.8%, respectively in PHS group of chickens due to PHS.

**Table 3:** The values of thyroid hormones and heart index in chickens.

Target	n	Control	PHS	*P* value
21 d				
T3 (µg/dL)	12	360.8 ± 24.9	577.7 ± 70.7^1^	0.018
T4 (µg/dL)	12	314.6 ± 46.1	352.2 ± 34.7	0.513
RV:TV	12	0.22 ± 0.004	0.28 ± 0.007^1^	0.000
35 d				
T3 (µg/dL)	12	318.2 ± 16.5	472.1 ± 67.2^1^	0.037
T4 (µg/dL)	12	212.8 ± 13.7	248.7 ± 32.2	0.334
RV:TV	12	0.21 ± 0.009	0.36 ± 0.012^1^	0.000

Values are means ± SE.

Abbreviations: n, number of broilers; PHS, pulmonary hypertension syndrome.

1 Significant difference between treatments in each time (*P* < 0.05).

### Thyroid Hormones

Plasma T3 concentration was significantly higher in the PHS group of chickens than in the control one (*P <* 0.05; Table 3). The amounts of this increase were 60.1% at 21d and 48.4% at 35 d of age. Cold stress resulted in no change in the plasma T4 concentration in 2 groups of chickens at 21 and 35 d.

### Relative Expression of Cardiac Genes

**Figure 2:**
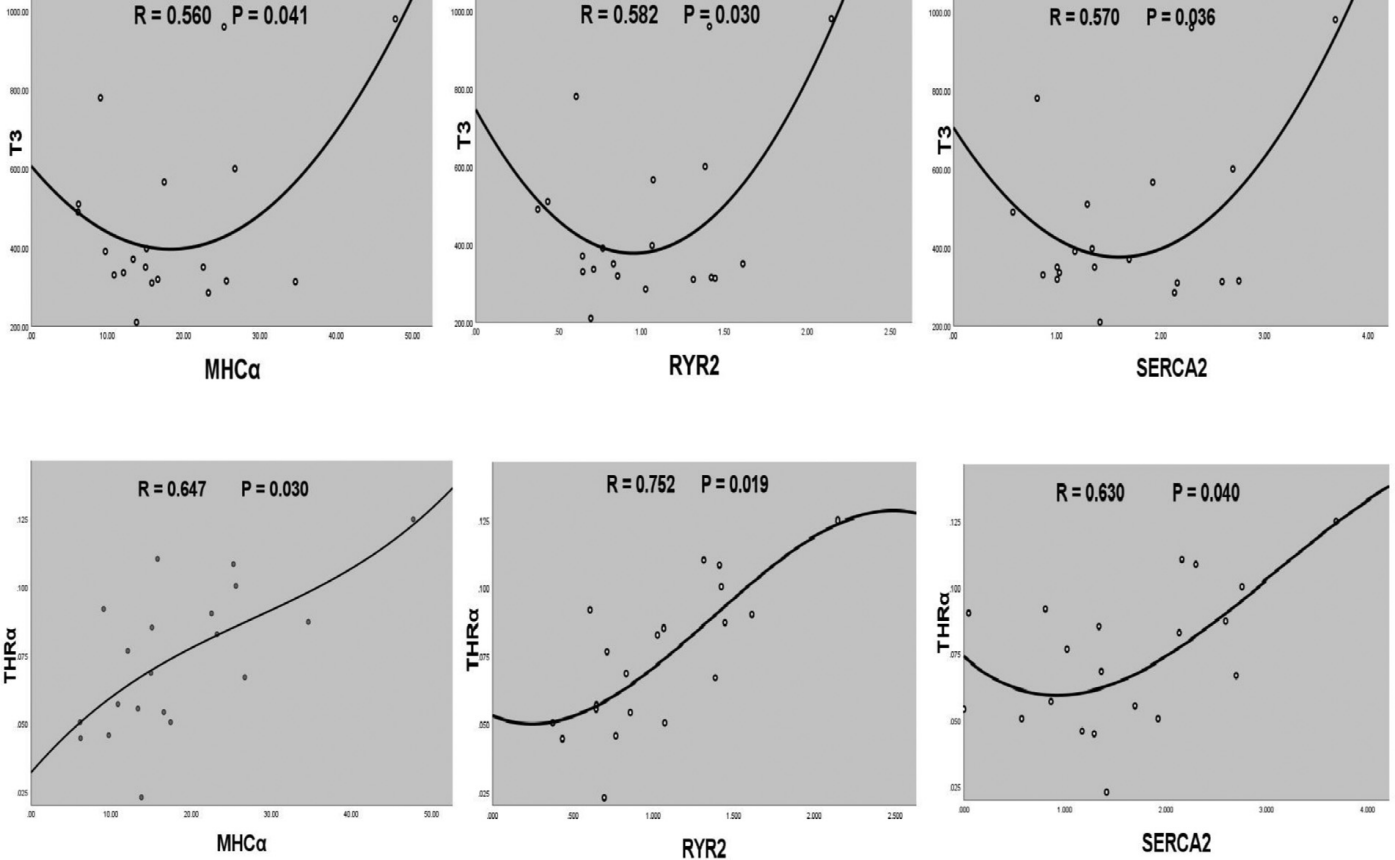
Graphs of regression analysis between MHCα, RYR2, SERCA2 mRNAs and T3, THRα in chickens at 21 days of age.

Real-time PCR results of ACTC1, MHCα, MHCβ, RYR2, SERCA2, THRα, THRβ, and troponin I genes are shown in Table 4. The transcript of YWHAZ as housekeeping gene was detected in all heart samples. The mentioned genes were expressed in the right ventricle of control and PHS groups of broilers at 21 and 35 d of age. The relative expression (target/ YWHAZ) of MHCα, RYR2, SERCA2, and THRα genes in the right ventricle tissues was higher in PHS group of broilers than control group at 21 d (*P* < 0.05).The amounts of this increase were 83.7%, 50%, 90.9%, and 50%, respectively in the mentioned genes. The expression of ACTC1, MHCβ, THRβ, and troponin I did not differ at 21d. The levels of mRNA for MHCα, RYR2, and SERCA2 genes exhibited a significant positive correlation with THRα mRNA and T3 at 21 d (Figure 2). The relative expression of ACTC1, MHCα, MHCβ, RYR2, SERCA2, troponin I and THRα genes in the right ventricle did not differ between control and PHS groups of broilers at 35 d (*P* > 0.05) while the expression of THRβ gene was higher (92.6%) in PHS groups of broilers than control at 35 d (*P* < 0.05)

**Table 4:** Relative gene expression (target/ YWHAZ) in the right ventricle of chicken heart.

Target	n	Control	PHS	*P* value
21 d				
ACTC1	12	22.5 ± 2.48	21.3 ± 1.93	0.705
MHCα	12	12.9 ± 1.58	23.7 ± 3.61^1^	0.014
MHCβ	12	0.004 ± 0.000	0.005 ± 0.001	0.865
RYR2	12	0.82 ± 0.020	1.23 ± 0.191^1^	0.037
SERCA2	12	1.1 ± 0.22	2.1 ± 0.28^1^	0.008
THRα	12	0.06 ± 0.007	0.09 ± 0.007^1^	0.015
THRβ	12	0.74 ± 0.094	0.69 ± 0.078	0.671
Troponin I	12	0.04 ± 0.008	0.04 ± 0.005	0.551
35 d				
ACTC1	12	18.4 ± 2.60	21.3 ± 3.35	0.513
MHCα	12	20.5 ± 4.82	18.6 ± 3.91	0.811
MHCβ	12	0.003 ± 0.001	0.004 ± 0.001	0.574
RYR2	12	1.3 ± 0.34	1.6 ± 0.33	0.477
SERCA2	12	1.8 ± 0.46	1.8 ± 0.38	0.984
THRα	12	0.07 ± 0.010	0.07 ± 0.011	0.959
THRβ	12	0.54 ± 0.099	1.04 ± 0.145^1^	0.014
Troponin I	12	0.03 ± 0.004	0.03 ± 0.003	0.883

Values are means ± SE.

Abbreviations: n, number of chickens; PHS, pulmonary hypertension syndrome.

1 Significant difference between treatments (*P* < 0.05).

## DISCUSSION

Cold stress is a factor that increases the rate of metabolic activity and, hence, the requirement of oxygen. A higher metabolic rate is associated with increased secretion of thyroid hormones. T3 is the main metabolic stimulating hormone (Kioukia-Fougia et al., 2002).

In the present study, we evaluated the plasma concentration of thyroid hormones and gene expression of its receptors (THRα and THRβ) and many cardiac proteins related to muscle contraction in two periods of age (21 and 35 d). In several studies, the positive correlation between low temperature and amount of thyroid hormones has been determined that the amounts of thyroid hormones (especially T3) were differed in order to cold severity, period of cold exposure, individual differences and metabolic adaptation rate in the birds (Wideman 2000; Luger et al., 2001; Balog et al., 2003). It has been reported that long exposure to low temperature in the birds causes compensatory changes in the cardiovascular system to accommodate oxygen needs and to adapt them to cold environment that sometimes lead to decrease of plasma thyroid hormones (Luger et al., 2001). However, many broiler chickens more often are not adapted to long exposure of cold and then showed the signs of PHS and ascites. In our study, cold stress resulted in the increased concentration of peripheral T3 in 21 and 35 d. This increase of T3 was more in 21 d than 35 d. In addition, the gene expression of heart THRα (as the most important receptor of T3) considerately increased in 21 d. it has been reviewed that high affinity of T3 for THRα and its low affinity for plasma proteins make T3 more powerful to act in the cells (Hulbert, 2000). Then, it would be predictable that effects of thyroid hormones in the heart were stronger in the age of 21 d than 35 d. In 35 d, the gene expression of THRβ only increased. The previous study showed that this receptor mediates limitary function in the heart and play predominantly a role in the control of heart rate (chronotropic effect) (Luger et al., 2001). Therefore, it seems that the heart (right ventricle) is severely impressed by T3 in 21d. This effect was properly observable in the gene expression of other contractile elements such as MHCα, SERCA2, and RYR2. The positive correlation between T3/THRα and these 3 genes was also confirmed. It has been determined that the increase of these elements potentiates the contractile force of cardiac musculature (inotropic effect) and leads to early cardiac (right ventricle) hypertrophy (Luger et al., 2001; Zarain-Herzberg 2006; Zou et al., 2011). The elevated RV:TV ratio is also an evidence for this early hypertrophy in the PHS group of chickens at 21 d. According to Wideman (2001), chickens with this amount of RV:TV (0.28) don't indicate PHS whereas Khajali and Khajali (2014) suggested that the RV:TV over 0.25 is as index for PHS and ascites. However, the RV:TV ratio in the range of 0.25 to 0.29 that shows the early right ventricular hypertrophy, could be the beginning of PHS and transition phase from adaptive hypertrophy to right ventricular failure (Chin et al., 2005), then, it may be known as “developmental PHS”.

It is noted that the mentioned genes in this study are responsive to thyroid hormones. In 35 d of age, despite of higher concentration of plasma T3 in PHS group of chickens, the expression of those genes did not change, that it could be due to no change in its receptor (THRα). Then, these data suggest that the action of thyroid hormones on myocardiocytes is dependent to both their plasma concentrations and receptor density as reported by Tavi et al. (2005).

In this study, the relative weight of right ventricle was greater in 35 d than 21 d, and the PHS mortality also occurred more in fifth week of rearing with severe signs of congestive heart failure. It has been reported that high concentration of thyroid hormones chronically could activate catabolic reactions in the cardiac cells and result in the deterioration of contractile proteins (Osuna et al., 2017). Hence, the higher weight of right ventricle at 35 d may be associated with its dilation (Khandwala, 2004).

The amount of troponin I, ACTC1, MHCβ transcripts did not change in the present study during cold stress and PHS in the broilers with hypertrophic heart or congestive heart failure whereas in the mammals, the regulation of these genes was strongly influenced (Kahaly and Dillmann, 2005). The reason for this controversy is not known but it could be regarding to species differences that in part, changes PHS pathogenesis in the birds as reported by previous studies (Teshfam et al., 2006; Hassanpour et al., 2009; Hamal et al., 2010; Hassanpour et al., 2016a,b).

Pulmonary hypertension syndrome is known as a disease with multifactorial pathogenesis that thyroid hormones and their receptors could be one of its crucial factors (Hassanzadeh et al., 2014; Janwari et al., 2018). The data determined that cold stress could increase thyroid hormones and the gene expression of their receptor (THRα) in the pick of chicken growth (21 d) that they themselves elevates the expression of many genes related to contractile elements (MHCα, RYR2, and SERCA2), leading to right ventricle hypertrophy.
